# Temporal Pitch Perception of Multi-Channel Stimuli by Cochlear-Implant Users

**DOI:** 10.1007/s10162-025-00983-4

**Published:** 2025-03-28

**Authors:** Evelien de Groote, Olivier Macherey, John M. Deeks, Stéphane Roman, Robert P. Carlyon

**Affiliations:** 1 Cambridge Hearing Group, MRC Cognition and Brain Sciences Unit, University of Cambridge, 15 Chaucer Rd, Cambridge, CB2 7EF UK; 2 Laboratoire de Mécanique Et d’Acoustique, Aix Marseille Université, Centre National de La Recherche Scientifique, Centrale Méditerranée, 13453 Cedex 13, France; 3 Dept. of Pediatric Otolaryngology and Neck Surgery, Aix-Marseille Univ, 13005 Marseille, France

**Keywords:** Cochlear implants, Pitch perception, Temporal fine structure, Fine structure processing

## Abstract

**Purpose:**

To explore the feasibility of cochlear-implant (CI) processing strategies that aim to improve pitch perception by presenting information on the stimulus temporal fine structure (TFS) in low-frequency channels to the corresponding apical electrodes.

**Methods:**

Eight users of the MED-EL CI pitch-ranked stimuli consisting of isochronous pulse trains presented concurrently to the four most apical CI electrodes.

**Results:**

When the same rate was applied to all electrodes, pitch ranks increased with increasing rates up to 200–300 pulses-per-second (pps), consistent with previous research. Presenting rates of 100, 200, 300, and 400 pps to one electrode per rate produced a pitch rank between that of the 100- and 200-pps same-rate stimuli. The assignation of pulse rate to electrode did not have a consistent effect on pitch ranks. However, maximising the delay between pulses on the different electrodes generally produced higher pitch ranks compared to when the between-electrode pulse delay was very short.

**Conclusion:**

Our results show no evidence that listeners combine the rates of TFS applied to different channels so as to estimate the fundamental frequency but do show that pitch can be affected by between-electrode delays. We conclude that presenting different temporal patterns to adjacent electrodes is unlikely to produce a clear and robust pitch and propose an alternative method for conveying the F0 of complex sounds on multiple electrodes of a CI.

## Introduction

In healthy acoustic hearing, the pitch of a periodic sound is dominated by its lower-numbered harmonics, each of which excites neurons that innervate inner hair cells in a restricted range of the basilar membrane [1]. Listeners combine information on the frequencies of these “resolved” harmonics to derive a pitch corresponding to the fundamental frequency (F0). The pitch of resolved harmonics supports very fine discrimination of F0 and is robust to differences in the phase of the constituent harmonics [2]. Changes in the pitch of resolved harmonics may be based on the neural phase-locking to each component, to changes in the place-of-excitation, or to some combination of the two codes [3–6]. Pitch can also be derived from the pattern of beating between the higher-numbered “unresolved” harmonics, several of which excite overlapping groups of auditory nerve (AN) fibres. This “purely temporal” pitch is less salient than that produced by resolved harmonics but can support musical-interval discrimination [7]. Experiments, where a harmonic complex is filtered so as to contain only unresolved harmonics, reveal F0 discrimination thresholds that are about an order of magnitude higher than when resolved harmonics are present [2, 8]. Unlike the case for resolved harmonics, both discrimination thresholds and the pitch that listeners report can depend strongly on the phase relationship between unresolved harmonics [2, 8].

Pitch perception by cochlear-implant (CI) listeners is substantially worse than that of people with normal (acoustic) hearing (NH). One reason for this arises from the way speech is conveyed by the CI sound processor. Most processing strategies extract the envelope in each frequency region, which is then used to amplitude-modulate a fixed-rate pulse train applied to one of the implanted electrodes. Information on the frequencies of individual harmonics is largely absent because the fairly broad analysis filters, combined with current spread along the cochlea, blur the spectral representation of the signal, and because the removal of the signal’s temporal fine structure (TFS) means that phase locking to the individual harmonics cannot occur. The remaining information on F0 primarily arises from beating between harmonics that fall within each analysis filter, which is then converted to amplitude modulation of the fixed-rate pulse trains presented on the corresponding electrodes. The representation of F0 in the response of a single electrode is broadly similar to that produced acoustically by a harmonic complex that has been filtered to contain only unresolved harmonics, in that it provides a purely temporal code that depends strongly on the modulation pattern and hence on the relative phases of the different harmonics. The modulations are not always aligned across electrodes, and so current spread between electrodes may produce more complex neural responses that combine these unaligned modulations.

**Fig. 1:**
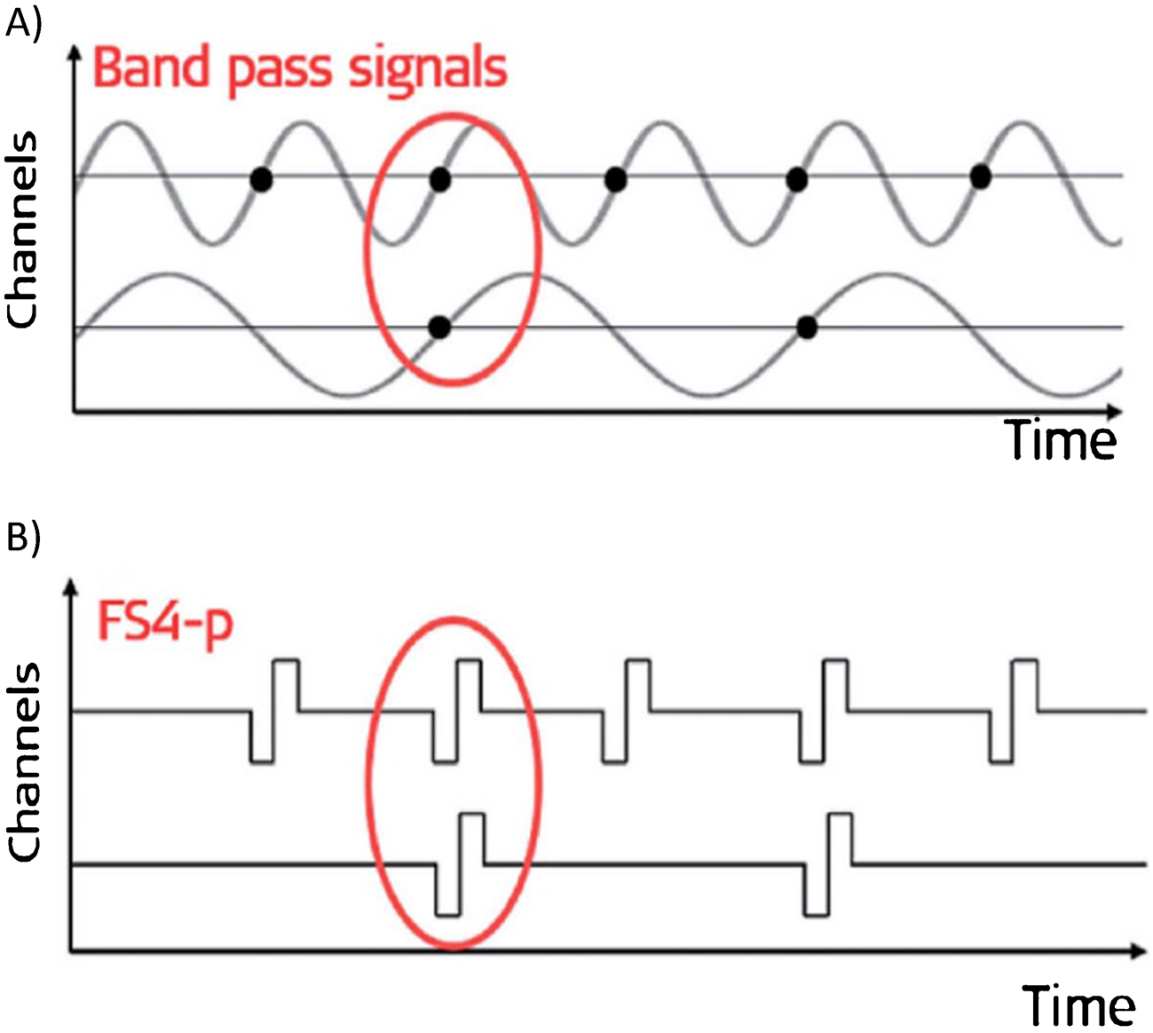
Schematic representation of two apical channels in the FS4-p processing strategy implemented by the MED-EL company. **A** The output of two apical analysis channels each of which passes a single component and where the two components are an octave apart. **B** The resulting pulse trains on the two channels. Note that **B** shows only one pulse per zero crossing instead of the short pulse-burst implemented in the real CI. Adapted slightly from Fig. 22 of Dhanasingh and Hochmair (2021), with permission

Pitch perception by CI listeners is also limited by biological and physical factors even when the processor is bypassed and idealised stimuli presented to one or more electrodes. For example, experiments that present a single-pulse-per-period (SPP) pulse train to one electrode show that some listeners can identify musical intervals, and that the most successful can detect changes in pulse rate of about 2–5%, but that performance varies substantially across listeners [9, 10]. Furthermore, there is an “upper limit” of about 300 pps above which increases in pulse rate do not produce an increase in pitch, again with substantial across-listener variation [11–13]. Nevertheless, it is likely important to present sufficient pitch information so as to produce the best possible pitch perception given the biological constraints, and there is considerable interest in improving the representation of pitch by CI processors. Attempts to achieve this goal fall broadly into one of two camps. One of these is to enhance and align the modulation patterns across electrodes and/or to extract the F0 and then selectively enhance modulations at that rate [14–18]. Experimental studies have revealed some improvements in pitch perception, but these methods have not been adopted by CI companies for clinical use. The other approach, which has been implemented in CI processors produced by two companies [19, 20], has been to present TFS information on the signal to a subset of apical electrodes. The present article focuses on this second approach, with particular reference to the class of “fine structure processing (FSP)” strategies implemented by the MED-EL company. These FSP strategies distinguish between apical and “remaining” channels. For each apical channel, a short burst of pulses is presented to the corresponding electrode at the zero-crossings in the analysis channel output (Fig. 1). The remaining channels are stimulated using a traditional strategy based on Continuous Interleaved Sampling [“CIS”: 21], whereby a fixed high-rate pulse train is modulated by the corresponding envelope. The output of a single channel to a low-frequency sinusoid will therefore consist of a pulse-burst that repeats at the frequency of the sinusoid, not dissimilar to the SPP pulse trains used to produce “optimal” pitch perception in experiments that bypass the processor.

The present study uses simplified stimuli to explore the pitch of complex sounds processed via an FSP type of strategy and was inspired by the psychophysical literature on temporal pitch perception both by CI and NH listeners. This literature reveals two basic requirements that, we argue, are important for FSP strategies to convey reliably the perception of the pitch of complex sounds. One of these arises from the fact that TFS strategies apply a different temporal pattern to each electrode (Fig. 1B), and so CI listeners would need to combine the TFS applied to each electrode to estimate the fundamental frequency (F0) of a sound. Psychophysical studies of temporal pitch perception by CI listeners usually apply the same rate to one or more electrodes, so it is not known how or even whether they perform this between-channel combination. Indeed, NH users are unable to extract the “missing fundamental” from pulse trains or modulated tones having rates of multiples of F0 and presented to different frequency regions, casting doubt on whether CI listeners would be able to perform an analogous task [22, 23]. However, those NH experiments presented stimuli to mid- and high-frequency regions so as to avoid any resolved harmonics, and so it is not known whether the brain could perform this calculation for stimuli that excite the apex of the cochlea.

Our second requirement arises from the fact that spread-of-excitation between electrodes may cause neurons to respond to the combined pulse pattern from multiple channels, leading to a complex temporal neural response that may be affected by frequency-dependent phase distortion arising from e.g. reverberation [24]. Hence, it is important for a TFS-based strategy that pitch judgements are unaffected by the relative timing of pulses applied to different channels.

The main part of the present study tests the above two requirements using a simplified simulation of the output of a TFS-conveying channel on the four most apical electrodes of the MED-EL CI. We also test whether pitch is dominated by the pattern of stimulation applied to either the most-apical or most-basal stimulated electrode, which might be less susceptible to between-electrode interference than is the case for the two “inner” electrodes. This part of the study involves pitch comparisons between stimuli that differ in the temporal patterns applied concurrently to the four electrodes and in the timing differences between those patterns. The goal is to probe multi-electrode pitch perception in idealised conditions and to discuss the implications of the results for pitch perception in more realistic listening conditions with present and future TFS-based strategies, rather than to exactly replicate FSP strategies as clinically implemented. As a precursor to the main part of the study, we also asked listeners to rank the pitches of the four electrodes presented in isolation, so as to check that this “place pitch” increased monotonically with increasing basal stimulation and to identify any exceptions to this trend.

## Methods

### Participants and General Methods

Eight participants (two women and six men aged 53–83 years) took part, two of whom (MED05 and MED06) were tested in Cambridge (UK), and six of whom were tested in Marseille (France). All were postlingually deaf and had been using their MED-EL device for at least 2 years at the time of the experiment. In the five bilaterally implanted participants, the longest-implanted side was tested. Except for participant MED06, all participants were implanted with either the longest (31.5 mm) or the second-longest (28.0 mm) electrode array manufactured by MED-EL. Participant MED06 received a 24-mm array to preserve low-frequency residual hearing. All participants were fitted with one of MED-EL’s FSP strategies for their daily (clinical) use. Table 1 shows the participants’ demographic and hearing-related characteristics, as well as their device specifications.

**Table 1:** Details of participants who took part in the experiments

Participant	Age (years)	Sex	Aetiology	Reported duration of profound HL (years)	Bilateral CI	Implant experience (years)	Tested side	Electrode array type	Active stimulation range (mm)	Deactivated electrodes	FSP strategy	*N* runs
M004	83	M	Unknown	10	Y	15	Right	Standard	31.5	/	FSP	5
M014	67	M	Autoimmune disease	1	Y	10	Right	FLEX28	28.0	/	FS4-p	5
M030	53	F	Otosclerosis	20	Y	6	Right	FLEX28	28.0	12	FS4	7
M031	74	M	Ototoxicity	11	N	7	Left	Standard	31.5	12	FS4	7
M032	62	M	Temporal bone fracture	1	Y	4	Right	FLEX28	28.0	/	FS4	7
M035	63	M	Meniere	28	Y	2	Left	FLEXSOFT	31.5	/	FS4	7
MED05	66	F	Unknown	19	N	11	Right	FLEX28	28.0	12	FS4	6
MED06	75	M	Genetic	35	N	18	Left	Medium	24.0	11,12	FSP	5

Note: The duration of hearing loss (HL) refers to the pre-implantation period. The tested side in bilaterally implanted participants was always the longest-implanted side. *N* runs refers to the number of repetitions of the rate-pitch ranking procedure that participants did. Fine-structure processing (FSP) strategy refers to the FSP strategy that participants were using on a daily basis

During the experiments, participants replaced their external speech processor with a coil connected to a computer via the MAX Programming Interface (MED-EL, Innsbruck). Stimulus presentation was controlled via interfaces programmed in MATLAB R2020b (Mathworks, Natick, MA, 2010) that used low-level routines provided by MED-EL. All stimuli were checked using a digital oscilloscope prior to data collection. Impedances were measured with the clinical fitting software MAESTRO 9.0 at the beginning and end of each session to ensure that all stimulation levels were within the compliance levels of the device. All stimulation was in monopolar mode, which is the only method supported by MED-EL CIs.

All participants provided informed consent and were compensated for their time.

### Place-Pitch Ranking

Place-pitch ranking was only performed for MED05 and MED06, as these data were available for all other participants who had participated in the study by de Groote et al. [25]. Electrical stimuli were 400-ms pulse trains presented at a rate of 80 pulses per second (pps). Biphasic pulses consisted of a 40-µs cathodic phase followed after an 8-µs inter-phase gap by an equal-duration and equal-amplitude anodic phase.

First, most comfortable levels (MCLs) were determined on individual electrodes (e1 to e4) for each participant using loudness scaling. Participants indicated the loudness of stimuli on a chart with loudness marked on a scale from “0 – No audible sound” to “10 – Too loud”. Participants were asked to indicate “1 – Just noticeable” at the first instance they heard a sound and to indicate their perceived loudness for each subsequent sound. Stimulus levels were increased until the level corresponding to a perceived loudness of “7 – Comfortable but Loud” was reached, after which the stimulus level was decreased. All participant responses were recorded. The MCL was defined as the midpoint of stimulus levels that the participant indicated as “6 – Most Comfortable”.

After loudness scaling, place-pitch ranking was assessed using the optimally efficient MidPoint Comparison [“MPC”, 26] procedure. In the standard version of this procedure, participants make a series of two-interval forced-choice comparisons (“Which sound has the highest pitch?”) without feedback: the two stimuli to be compared on each trial are selected based on the results of the previous trial so as to minimise the total number of comparisons. The inter-stimulus interval was always 800 ms. We used the “best of three” modification implemented by Adel et al. [27] after Levitt and Rabiner [28], in which comparisons are initially presented twice, with the order of the stimuli randomised each time. Participant responses in those two trials were then compared. In case of consistent responses on both trials, the procedure continued with the next comparison. In case of inconsistent responses on the two trials, a third comparison was presented, and the trial was scored according to the stimulus judged higher on two out of the three trials. This procedure was run 5 times (or 10 times for participant M004), each with the stimuli entered into the ranking procedure in a different random order. For each electrode, the mean pitch rank and standard deviation were calculated across those runs.

### Rate-Pitch Ranking

**Fig. 2:**
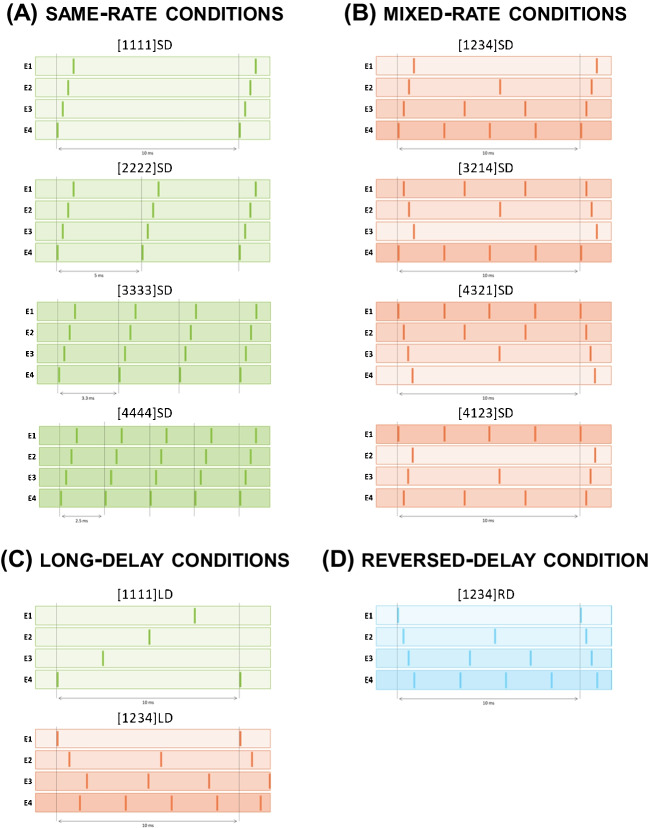
Schematic representation of the 11 multi-electrode stimuli used in the rate-pitch ranking experiment. The shaded horizontal sections represent different electrodes (e1 to e4), and small vertical bars represent biphasic pulses. Green colours indicate the same-rate conditions (**A**) that put the same rate on each electrode. Orange colours indicate the mixed-rate conditions (**B**) that put different rates on each electrode. Blue colours (**D**) indicate the reversed-delay condition. The long-delay conditions are shown, shaded by the appropriate colour, in **C**. For each stimulus, increasingly darker shades indicate increasingly higher pulse rates. The arrows indicate one period of the multi-electrode stimulus

The rate-pitch ranking task included 11 multi-electrode stimuli that differed with respect to the allocation of pulse rates across the four most apical electrodes, the order of stimulation, and the inter-electrode delays. Table 2 presents a detailed overview of the multi-electrode stimuli used, and Fig. 2 provides a visualisation of the same stimuli. All multi-electrode stimuli were trains of symmetric biphasic pulses and had a duration of 400 ms. Pulses consisted of a 40-µs cathodic phase followed after an 8-µs inter-phase gap by an equal-duration and equal-amplitude anodic phase. Individual electrodes were stimulated sequentially with either a short (SD) or long (LD) inter-electrode delay. The SD was always set to 100 µs across different stimulation conditions. It allowed the pulses on different channels to be nearly synchronous whilst reducing (although not necessarily eliminating) charge interactions between temporally adjacent pulses [29, 30, 31]. The LD was set to maximise the inter-pulse intervals (IPI) and to produce (as much as possible) stimuli with evenly spread pulses.

**Table 2:** Overview of multi-electrode stimuli. Condition names refer to the pulse rates presented to e1–e4 (where 1 = 100 pps, 2 = 200 pps, etc.) and the inter-electrode delay

Condition number	Condition name	Pulse rate, pps (start time offset, ms)			
		e1	e2	e3	e4
1	[1111]SD	100 (0.3)	100 (0.2)	100 (0.1)	100 (0)
2	[2222]SD	200 (0.3)	200 (0.2)	200 (0.1)	200 (0)
3	[3333]SD	300 (0.3)	300 (0.2)	300 (0.1)	300 (0)
4	[4444]SD	400 (0.3)	400 (0.2)	400 (0.1)	400 (0)
5	[1234]SD	100 (0.3)	200 (0.2)	300 (0.1)	400 (0)
6	[3214]SD	300 (0.1)	200 (0.2)	100 (0.3)	400 (0)
7	[4321]SD	400 (0)	300 (0.1)	200 (0.2)	100 (0.3)
8	[4123]SD	400 (0)	100 (0.3)	200 (0.2)	300 (0.1)
9	[1111]LD	100 (7.5)	100 (5.0)	100 (2.5)	100 (0)
10	[1234]LD	100 (0)	200 (0.63)	300 (1.67)	400 (1.25)
11	[1234]xRD	100 (0)	200 (0.1)	300 (0.2)	400 (0.3)

*SD* short delay, *LD* long delay, *RD* reversed delay, *pps* pulses per second

Conditions 1–4 represent same-rate stimulation conditions in which the four most apical electrodes are stimulated at the same pulse rate of either 100, 200, 300, or 400 pps with short basal-to-apical inter-electrode delays of 100 µs (Fig. 2A). The leading electrode was always the most basal channel (e4). We expected the same-rate conditions to elicit unambiguous pitch percepts and to serve as a comparison for the pitch elicited in other stimulation conditions.

In conditions 5–8, pulse rates of 100, 200, 300, and 400 pps were each assigned to one electrode (Fig. 2B). These “mixed-rate” conditions differed only in the assignation of rate to electrodes and in the relative timing between the pulses on different electrodes.

Condition 5 ([1234]SD) is the harmonically related mixed-rate multi-electrode stimulus of main interest and represents a simple approximation of MED-EL’s FS4 strategy. This stimulus is roughly analogous to a situation in which the input is a sound consisting of equal-amplitude harmonics, whereby the first four harmonics correspond to the centre frequencies of electrodes e1 to e4, and where the analysis filters are sufficiently sharp to pass only one harmonic each. (Note that this does not correspond to the frequency-to-electrode mapping used clinically, nor to the real-world situation where the relative amplitudes of the harmonics depend on the spectral shape of e.g. the speech token being uttered.) In this condition, the four most apical electrodes e1 to e4 are stimulated at 100, 200, 300, and 400 pps, respectively, with short basal-to-apical inter-electrode delays of 100 µs. Condition 6 ([3214]SD) examines whether the pitch of a multi-electrode stimulus is dominated by the rate presented to the most-apical electrode. This might occur because that electrode excites a wide range of apical neurons, whose responses are uncorrupted by stimulation of a more proximal electrode, and/or because of evidence that temporal coding is more accurate at the apex of the cochlea [e.g. 32, but see 25]. If so, then [3214]SD should have a higher pitch than [1234]SD because the rate on e1 is 300 pps instead of 100 pps. Similarly, a comparison between conditions 7 ([4321]SD) and 8 ([4123]SD) examines whether the pitch of a multi-electrode stimulus is dominated by the rate presented to the most basal electrode, which may excite a wide range of even-more basal neurons whose responses are unaffected by a closer electrode. If it is, then [4321]SD should have a lower pitch than [4123]SD. To investigate the effects of the inter-electrode delay and the order of stimulation, various delay conditions were included in the procedure (Fig. 2C). Conditions 9 ([1111]LD) and 10 ([1234]LD) represent the LD counterparts of conditions 1 and 5. The offsets for [1111]LD were set so that e3 to e1 were increasingly delayed by one-fourth of a period relative to the first-stimulating channel e4. The offsets for condition [1234]LD between 100-, 200-, and 400-pps pulse trains were chosen so as to produce the largest minimum IPI for those three rates combined. The offset for the 300-pps pulse train was then varied so as to produce the largest minimum IPI between any two pulses. By comparing SD to LD stimuli, which were identical on all other stimulus parameters, we tested our second requirement, namely that the pitch of multi-electrode stimuli should be unaffected by the relative timing of the pulses on different channels. If this requirement is met, then the pitch of each LD stimulus should equal that of its SD counterpart. If, on the other hand, adjacent electrodes stimulate overlapping neural populations and neurons innervating the same cochlear region are excited by a complex mixture of different pulse rates originating from different electrodes, this might increase the composite temporal pitch of the LD compared to the SD stimulus [33]. Finally, condition 11 ([1234]xRD; Fig. 2D) tested whether changing the stimulation order of electrodes introduces place-of-excitation cues that may confound temporal pitch effects. This was of concern as pulses may partially mask the effect of subsequent pulses as a result of spread of excitation, especially when sequentially stimulating adjacent electrodes. It differs from the [1234]SD stimulus of condition 5 only in that the first pulse of each adjacent pair is applied to the more-apical instead of to the more-basal electrode—i.e. the stimulation order is apical-to-basal instead of basal-to-apical. If masking affects place pitch, then the pitch rank of [1234]xRD should be lower than that of [1234]SD.

Prior to the pitch judgements, the loudness of the multi-electrode stimuli was set in three steps. First, MCLs were determined for single-electrode stimulation using a subset of seven electrode-rate combinations consisting of all rates for electrode 2 and all electrodes for a rate of 200 pps, using the same loudness-scaling procedure as described above. MCLs for all other combinations of electrode (e1, e3, and e4) and rate (100, 300, and 400pps) were linearly interpolated in dB. Second, multi-electrode stimuli were constructed by fixing the relative rate-specific across-electrode level differences constant in dB, based on the MCLs measured for single-electrode stimulation in step 1. These stimuli were then loudness scaled, again using the same loudness-scaling procedure as described above. Third, all multi-electrode stimuli were then loudness balanced to a reference [2222]SD condition by presenting them in pairs and using an adjustment paradigm. In this paradigm, the level of the first sound was always fixed at MCL, whereas the start level of the second sound was randomly set to the level corresponding to loudness “4 – Comfortable but Soft” or “7 – Loud but Comfortable”. Different start levels were used on different runs to discourage participants from repeating their responses. Participants adjusted the level of the second sound themselves by pressing one of six buttons labelled “-”, “- -”, and “- - -” to make the second sound increasingly softer and “ + ”, “ + + ”, and “ + + + ” to make the second sound increasingly louder. The different buttons corresponded to 1-, 3-, and 6-BIT steps (in the RIB experimental software, 1 BIT corresponds to current steps of 1.18, 2.36, 7.71, and 9.45 µA for ranges 0 (0–150 µA), 1 (0–300 µA), 2 (0–600 µA), and 3 (0–1200 µA), respectively. We used the lowest possible range for each participant that allowed all stimuli to be presented at a level at or below MCL; this was always range 1 or 2. Each time one of the buttons was pressed, the stimulus pair was presented again until the two sounds were judged as equally loud. Each pair of stimulation conditions was balanced four times (with the order of fixed and adjustable sound switched halfway). The level of the non-reference stimulation condition was changed to match the loudness of the MCL of the reference [2222]SD condition.

Following MCL determination, pitch ranking was assessed using the MPC procedure with the best-of-three adaptation as described above. The MPC procedure was run 5 to 7 times each, depending on the participant, with the stimuli presented in a different random order each time. For each multi-electrode stimulus, the mean pitch rank and standard deviation were calculated across those runs. In addition, we repeated the rate-pitch ranking experiment with participants MED05 and MED06 with the sole modification of including two additional same-rate conditions with pulse rates of 50-pps and 71-pps on each channel. These measurements took place over one additional session for MED05 and two sessions for MED06 and consisted of five and eight pitch-ranking runs, respectively. These supplementary measures were performed as a check for the effects of stimulus range on pitch ranks [34] and will be considered in the Discussion.

## Results

### Loudness Balancing

Table 3 shows the loudness-balanced stimulus levels, defined as the current applied to electrode 1, for all participants and conditions. The mean levels are generally very similar across conditions, with the exception of the two long-delay conditions, where the levels were higher than their short-delay counterparts by 1.5 dB ([1111]LD vs [1111]SD) and 1.0 dB ([1234]LD vs [1234]SD), respectively. The greater loudness for the short-delay conditions could be due to charge summation between channels [30, 31] and/or to the greater output of a central “temporal window” when the interval between pulses that excite overlapping neural populations is short [35]. The difference was significantly larger for the same-rate ([1111]SD/LD) than for the mixed-rate ([1234]SD/LD) comparison (*t*(7) = 2.50, *p* = 0.04, effect size (Cohen’s *d*) = 0.88), consistent with the fact that, for the same-rate stimuli, every pulse is temporally adjacent to pulses on other channels (Fig. 1). Table 4 shows the size of the LD-SD difference for each participant and for the two conditions.

**Table 3:** The stimulus level (dB re 1 µA) presented on electrode 1 for every participant and condition, with mean data shown in bold in the bottom row

	**[1111]SD**	**[2222]SD**	**[3333]SD**	**[4444]SD**	**[1234]SD**	**[3214]SD**	**[4321]SD**	**[4123]SD**	**[1111]LD**	**[1234]LD**	**[1234]RD**
M004	51.6	51.1	51.0	50.8	51.4	50.8	50.5	50.9	52.5	52.0	51.4
M014	47.9	47.9	47.7	47.3	48.0	47.5	47.2	47.4	49.7	49.0	48.2
M030	46.0	45.8	45.8	45.8	46.4	45.5	45.2	45.1	46.8	47.4	46.4
M031	47.0	47.0	46.8	46.7	47.2	46.9	46.5	46.4	48.2	47.8	47.0
M032	46.4	46.4	46.6	46.6	46.7	46.6	46.6	46.6	48.3	48.3	46.6
M035	47.6	47.4	47.5	47.5	47.2	47.0	46.8	46.9	49.6	49.4	47.0
MED05	47.2	47.1	47.2	46.8	47.1	47.3	47.2	47.2	49.0	48.3	47.0
MED06	48.8	48.6	48.1	47.9	49.2	48.2	48.5	48.0	50.0	49.2	49.1
**MEAN**	**47.8**	**47.7**	**47.6**	**47.4**	**47.9**	**47.5**	**47.3**	**47.3**	**49.3**	**48.9**	**47.9**

**Table 4:** The difference (LD-SD, in dB) between each LD condition and its SD counterpart, with mean data shown in bold in the bottom row

	**[1111]**	**[1234]**
M004	0.9	0.6
M014	1.8	1
M030	0.8	1
M031	1.2	0.6
M032	1.9	1.6
M035	2	2.2
MED05	1.8	1.2
MED06	1.2	0
Mean	**1.6**	**1.0**

### Place-Pitch Ranking

**Fig. 3:**
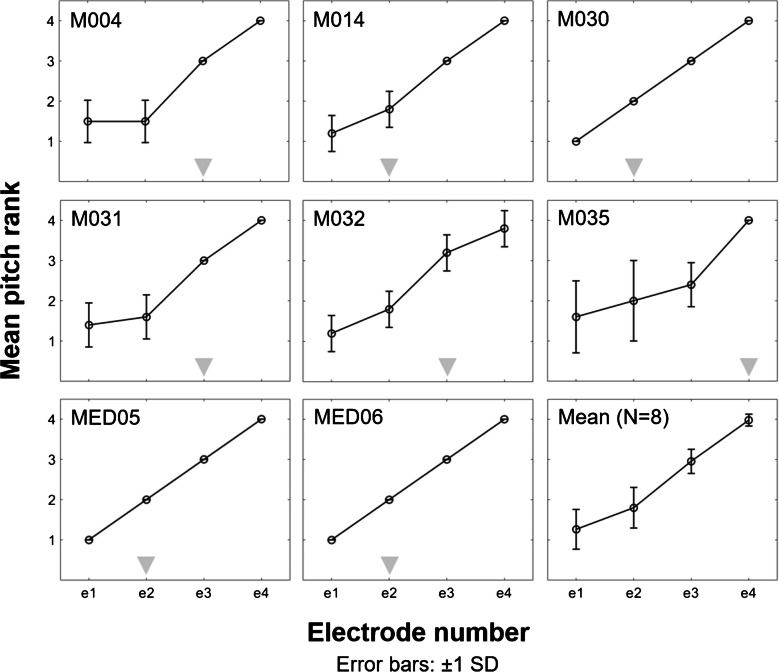
Mean place-pitch ranks and standard deviations (SD) obtained from five runs (or 10 for participant M004) of the midpoint comparison procedure for individual participants and averaged across participants (bottom right). For each participant, the grey triangle at the bottom of each graph indicates the closest electrode that produced a mean pitch rank higher than the mean pitch rank + 1 SD at e1

The mean place-pitch and standard deviations for individual participants and the average pitch ranks across participants are shown in Fig. 3. A pattern of monotonically increasing pitch ranks with increasing electrode number can be observed for most participants. However, at the most apical electrodes, some participants show a relatively flat pitch-rank function. This observation is consistent with poor apical place-pitch encoding in participants M004, M031, and M035. No pitch reversals, such as have been previously reported with the apical electrodes of the MED-EL device [36, 37], were observed.

### Rate-Pitch Ranking of Multi-electrode Stimuli

**Fig. 4:**
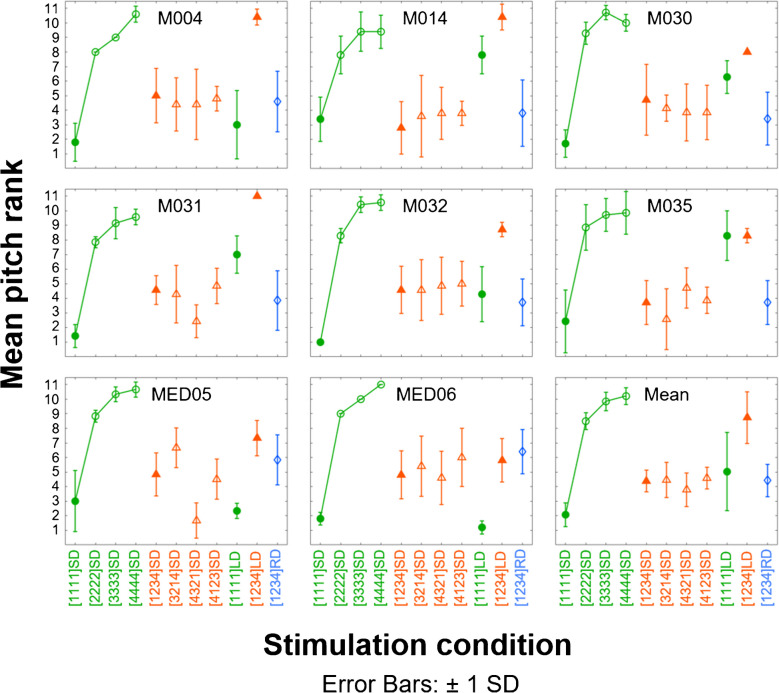
Mean rate-pitch ranks and standard deviations (SD) obtained from five to seven runs of the midpoint comparison procedure for individual participants and averaged across participants (bottom right). The same-rate stimuli are presented by circles, of which the short-delay (SD) conditions are joined by lines. The mixed-rate conditions are presented by triangles. Filled markers indicate the SD to long delay (LD) comparisons. The reversed-delay (RD) condition is marked by a diamond

Each of the first 8 panels of Fig. 4 shows one participant’s mean pitch rank and standard deviations for the 11 multi-electrode stimuli shown in Table 2. Group mean data are shown in the last (bottom right-hand) panel together with between-participant standard deviations. The ranks for conditions 1 to 4 are shown in green by circles joined by lines; the lines were added for clarity, but note that all stimuli were included in every pitch-ranking run. It can be seen that for these conditions, in which the same rate is applied to all electrodes, the pitch increases with increasing pulse rate for all participants, with a plateau starting at 200 or 300 pps depending on the participant. These data are broadly consistent with the comprehensive literature on the pitch of SPP pulse trains applied to single electrodes and are used below to aid the qualitative interpretation of the pitch ranks obtained in the other stimulus conditions, where different rates are applied concurrently to the different electrodes. Our quantitative analyses are confined to *t*-tests of the six comparisons outlined in the methods and that were the basis of our experimental design, and which are described in turn below. We divide the criterion significance levels of 0.05 and 0.01 by 6 to control for type 1 errors arising from these multiple comparisons, leading to corrected criteria of *p* = 0.0083 and *p* = 0.0017; to aid the reader, *p* values that meet these criteria are appended by single and double asterisks, respectively. Effect sizes (Cohen’s *d*) are given regardless of whether the *p* value is significant at the corrected level. Shapiro–Wilk’s tests on the participant-by-participant differences between the conditions to be compared in each *t*-test revealed no evidence of a significant deviation from normality in any case.

Our first comparison concerns the question of whether listeners hear the “fundamental frequency” of 100 Hz in condition 5 ([1234]SD; left-most filled orange triangle in each plot). Inspection of the individual data clearly shows that this was not generally the case, with all participants except M014 producing a pitch rank between that of the [1111]SD and [2222]SD stimuli. The mean pitch rank for participant M014 was close to that of the [1111]SD stimulus but with slightly larger standard deviations. At the group level, a *t*-test between the pitch ranks of [1234]SD and [1111]SD showed that the [1234]SD had a significantly higher pitch (*t*(7) = 4.65, *p* = 0.002*, *d* = 1.6).

Our next two comparisons test whether the pitch of multi-rate stimuli is dominated by the stimulation pattern applied to either the apical or basal electrode. These two comparisons are between [1234]SD vs. [3214]SD to test for apical-electrode dominance and between [4321]SD vs. [4123]SD to test for basal-electrode dominance. Inspection of the individual data (pairs of adjacent orange triangles in centre of each plot) shows that the two stimuli comprising each comparison had very similar pitch ranks for all participants, with the exception of M031 and MED05 for whom [4123]SD had a higher pitch rank than [4321]SD, as would be expected if the pulse rate on electrode 4 had a dominant effect on pitch. Even for these participants, the dominance could not have been complete, as the pitch rank of [4123]SD (right-most open orange triangle) was substantially lower than that of [3333]SD.

Comparisons four and five address the second requirement laid out in the Introduction, namely that pitch should not depend on the relative timing of the pulses applied to the different electrodes. They do so by comparing the pitch ranks of the SD vs. LD versions of [1234] and of [1111] and are indicated by filled symbols in each plot. For the multi-rate stimulus ([1234]; compare filled orange triangles), all participants, with the exception of MED06 and possibly MED05, assigned higher pitch ranks to the LD than to the SD version, and this difference was highly significant at the group level (*t*(7) = 5.79, *p* = 0.0007**, *d* = 2.0). The pitch ranks for the same-rate stimulus ([1111]; filled green circles) were often (and on average) higher for the LD version, but with the LD-SD difference varying across participants and being absent for MED05 and MED06, and also possibly for M004. At the group level, the difference was not significant at the corrected 5% level (*t*(7) = 3.15, *p* = 0.016, *d* = 1.1).

Finally, to test for the possibility that masking affected place pitch and hence pitch ranking, we compared [1234]SD with [1234]RD (right-most symbol shown by blue circle). Pitch ranks for these two stimuli were similar for all participants and, not surprisingly, did not differ significantly at the group level (*t*(7) = 0.12, *p* = 0.911, *d* = 0).

## Discussion

The Introduction described two requirements that, we argue, are important for FSP strategies to convey reliably the pitch of complex sounds. Our results show that, for the simplified stimuli used here, neither requirement is met: listeners do not typically report a pitch corresponding to the F0, and pitch estimates can be substantially affected by between-channel timing differences. The following discussion relates these findings to the existing literature, considers possible reasons for between-listener differences, describes possible limitations of our simplified approach, and proposes a more robust method for using timing cues to convey pitch.

### Comparison to Previous Findings

Figure 4 shows that pitch ranks for our same-rate stimuli increased markedly and consistently with increases in pulse rate from 100 to 200 pps per channel, but less so with further increases to 300 and (especially) 400 pps. This general pattern of results is broadly consistent with a large literature on the upper limit of pitch for SPP pulse trains presented to a single electrode [11–13] and to multiple electrodes [38–41]. Recently, we measured pitch ranking over a wide range of rates (80–981 pps) for a stimulus in which the four apical electrodes of the MED-EL device were stimulated simultaneously, rather than with the 100-µs offset between adjacent channels applied in the present study [25]. The upper limit in the article by de Groote et al. [25] was estimated by fitting a broken-stick function to the pitch-rank data. Six participants (M004, M014, M030, M031, M032, and M035) also took part in the present study. They showed upper limits in the previous study of between 335 and 686 pps, which are generally higher than the rates above which pitch ranks appear to asymptote here. This might suggest that rate discrimination is better with simultaneous than with interleaved stimulation. However, it is not straightforward to compare pitch ranks obtained with different ranges of stimuli and in a different context, and so we would refrain from drawing this conclusion in the absence of a direct comparison. In addition, an inspection of the data in de Groote et al. [25] reveals some flattening of the pitch-rank function around 335 pps in all six listeners who also took part in the present study, even when the upper limit derived from the broken-stick fit was substantially higher than 335 pps.

The data presented in Fig. 4 show that between-channel timing differences can have substantial effects on pitch ranks. The size of this effect varied across listeners, but it was highly significant at the group level for the multi-rate comparison ([1234]SD vs [1234]LD, d = 2.0). Although the comparison for the 100-pps same-rate stimuli ([1111]SD vs [1111]LD) did not reach significance at the Bonferonni-corrected level, the pitch rank for the [1111]LD stimulus for participants M014, M031, and M035 was close to that for the [2222]SD stimulus—i.e. manipulating the between channel delay had an effect similar to doubling the pulse rate. The effect of across-channel timing on pitch has been previously studied using pairs of pulse trains presented to the same or different electrodes and by implementing a paradigm based on a method introduced by McKay and McDermott [33] in a study with five participants implanted with the Cochlear CI. Macherey and Carlyon [42] asked six users of the Advanced Bionics and Cochlear CI to pitch-rank 2-channel stimuli, each of which consisted of a train of pulses that had the same rate in each channel and with a between-channel delay that was either very short (< 400 µs) or equal to half the period. These two delays are broadly comparable to those in our [1111]SD and [1111]LD conditions. The pulse rate per channel ranged from 92 to 516 pps in half-octave steps, making it possible to estimate the rate of an SD stimulus having the same pitch as each LD stimulus. They found that, for most listeners, the pitch of an LD stimulus of a given rate was very close to that of an SD stimulus of the same rate, even when the two pulse trains were presented to adjacent electrodes—that is, the across-channel delay had only a small effect on pitch. A similar finding was obtained by Griessner et al. [43] in an experiment that presented pairs of pulse trains to two adjacent apical electrodes in ten participants implanted with the MED-EL CI. Hence, with the *caveat* that, as with almost all CI experiments, effects can differ between groups of listeners, our results—at least in the multi-rate condition—show a larger effect of across-channel timing than has been reported previously with same-rate stimuli presented to two electrodes. One possible reason for this is that neurons may be activated by more than two electrodes with our stimuli and that this increases the temporal complexity of the neural responses compared to the case where only two electrodes are stimulated. Alternatively, greater across-channel interactions between pulses, due either to charge summation or refractory effects, might change the shape of the neural excitation pattern.

### Pitch-Ranking Variability Between and Within Listeners

As noted above, the size of some of the effects on temporal pitch ranking, observed at the group level, differed across listeners. In particular, participants MED05 and MED06 showed no effect of across-channel timing on the pitch ranks of the [1111] SD vs LD stimuli and also showed smaller effects than other participants for the [1234] SD vs LD comparison. Participant MED05 was unusual in that her pitch rankings depended, for the multi-rate stimuli, on the assignment of rates to electrodes (conditions 5–8; orange symbols); her rank for [4321]SD was similar to that for [1111]SD and lower than for [1234]SD, [3214]SD, and [4123]SD. This pattern of results is consistent with the pitch ranks for MED05 being strongly influenced by the pulse rate applied to electrode 4 when that rate was 100 pps. As noted above, when e4 was stimulated at 400 pps as in [1234]SD, the pitch rank was lower than for [4444SD], suggesting that under those circumstances, the lower-rate stimulation on e1-3 influenced her pitch judgements. In contrast to the results for MED05, the pitch ranks for participant MED06 were similar for conditions 5–8, and so there was no evidence that his judgements were dominated by the rate applied to any one electrode. His pitch judgements may have been based on a combination of the separate rates applied to more than one electrode, unlike those of the majority of participants whose judgements were affected by between-channel delays. It is worth noting that both MED05 and MED06 showed excellent place-pitch ranking of the individual electrodes (Fig. 2), consistent with the idea that the effect of between-channel timing on pitch judgements depends on the extent of between-channel interactions and hence on spatial selectivity, which in turn affects pitch ranking. We counsel some caution, however, given the modest number of listeners tested and the fact that listener M030, who also showed excellent place-pitch ranking, showed between-channel timing effects similar to that in the group-averaged data.

The temporal pitch ranks shown in Fig. 3 differed not only between conditions but also between the individual runs for a given listener and condition. Inspection of the error bars in the figure indicates that these were generally larger for the multi-rate conditions (triangles) than for the same-rate conditions (circles). For example, the between-run variance, averaged across listeners, corresponded to standard deviations of 1.4, 0.8, 0.8, and 0.8 for the four same-rate SD conditions (1–4) on the left of each plot but 1.7, 2.0, 1.8, and 1.4 for the next four mixed-rate conditions, respectively. This suggests that the mixed-rate stimuli, whereby the temporal response is likely to differ between electrodes, had a less well-defined pitch than that conveyed by presenting the same temporal stimulus to each channel.

### Limitations

The stimuli employed here were not intended to reproduce exactly the pattern of stimulation that would be provided by a MED-EL FSP processing strategy in everyday life and differed from that pattern in several ways. Rather, they provide a simplified stimulus set that allows us to evaluate the processes by which CI listeners can—and, importantly, cannot—combine information from multiple electrodes to perceive the pitch of a complex sound.

One simplification is that we restricted stimulation to have either the same or harmonically related rates on each of the four electrodes stimulated. This was done in order to give the CI participants the best chance to hear a complex pitch. We have argued that acoustic pulse trains filtered to contain only unresolved harmonics provide a useful NH analogue of the perception of pulse trains by CI listeners, and experiments using those stimuli show that mixtures of inharmonically related pulse rates produce an unpleasant “crackle” percept without a clear pitch [44]. We therefore expect that, had we included inharmonically related rates, pitch perception would have been even worse. It is also possible that the outputs of some channels of an FSP strategy would be amplitude modulated at F0 due to beating between adjacent harmonics that fall within the passband of an analysis filter, and that this would have improved the perception of F0. However, this is arguably an envelope cue that would also be present in the output of a traditional CIS strategy, rather than reflecting a feature of the stimulus fine structure. We additionally note that the temporal fine structure at the output of a channel that passes (say) two harmonics with equal amplitude can be more complex than that occurring when it is dominated by a single harmonic. Another simplification was to present a single pulse per period instead of the short burst of pulses produced by MED-EL’s FSP strategies. We do not think that this would have degraded the pitch perception of the multi-rate stimuli studied here and cannot think of a reason why it would have done so. Finally, we note that our stimuli combined equally loud pulse trains from each electrode, roughly corresponding to a situation in which the harmonics of a complex sound had equal amplitudes and corresponded to the centre frequency of each channel. In a real situation, the amplitude of the pulses on each channel will vary with the spectral shape of the input, which for a speech sound such as a vowel will depend on the formant frequencies and hence on vowel identity. It is of course very important that perceived pitch does not, for a fixed F0, depend strongly on vowel identity, and it is a crucial feature of models of NH pitch perception that pitch is independent of spectral shape [e.g. “the case of the missing fundamental”; 45]. Our data do not tell us anything about the dependence of pitch conveyed by TFS strategies on spectral shape, but we do not think that variations in e.g. vowel identity would somehow allow listeners to combine the TFS applied to each electrode so as to provide a more robust estimate of F0, as in our first requirement.

A second potential limitation arises from the MPC pitch-ranking method used. Although this method is well-established in CI research, it does, as with all pitch-ranking and scaling methods, implicitly assume that listeners are responding along a single perceptual dimension (i.e. pitch). The procedure involves a series of forced-choice comparisons without feedback, and so responses were presumably based on a percept that listeners spontaneously interpret as pitch [cf. 46], but we did not include alternative tasks, such as melody identification, that may be more relevant for the perception of music. However, these considerations are arguably more pertinent to interpreting cases where participants reliably and consistently assign different ranks to two or more stimuli, and to the issue of whether doing so genuinely involves a difference in pitch, than to cases where a group of stimuli is generally assigned the same pitch rank. One of our two main questions concerns the pitch ranks of mixed-rate SD stimuli, which generally fall into the latter category, and for which there is no evidence of a percept equal to that produced when the F0 rate is applied to all electrodes ([1111]SD). Our second main question concerns the effect of increasing between-channel delays, which reliably increase pitch ranks, as has been observed in previous studies where the delays are applied to pulses interleaved on the same channel, and as would be expected in conditions where neurons respond to the composite pitch rate from two or more electrodes [33, 42, 43]. However, as with all pitch-comparison procedures, we cannot completely rule out the possibility that these delays affected some percept other than pitch but that nevertheless affected the pitch judgements that participants were instructed to make.

**Fig. 5:**
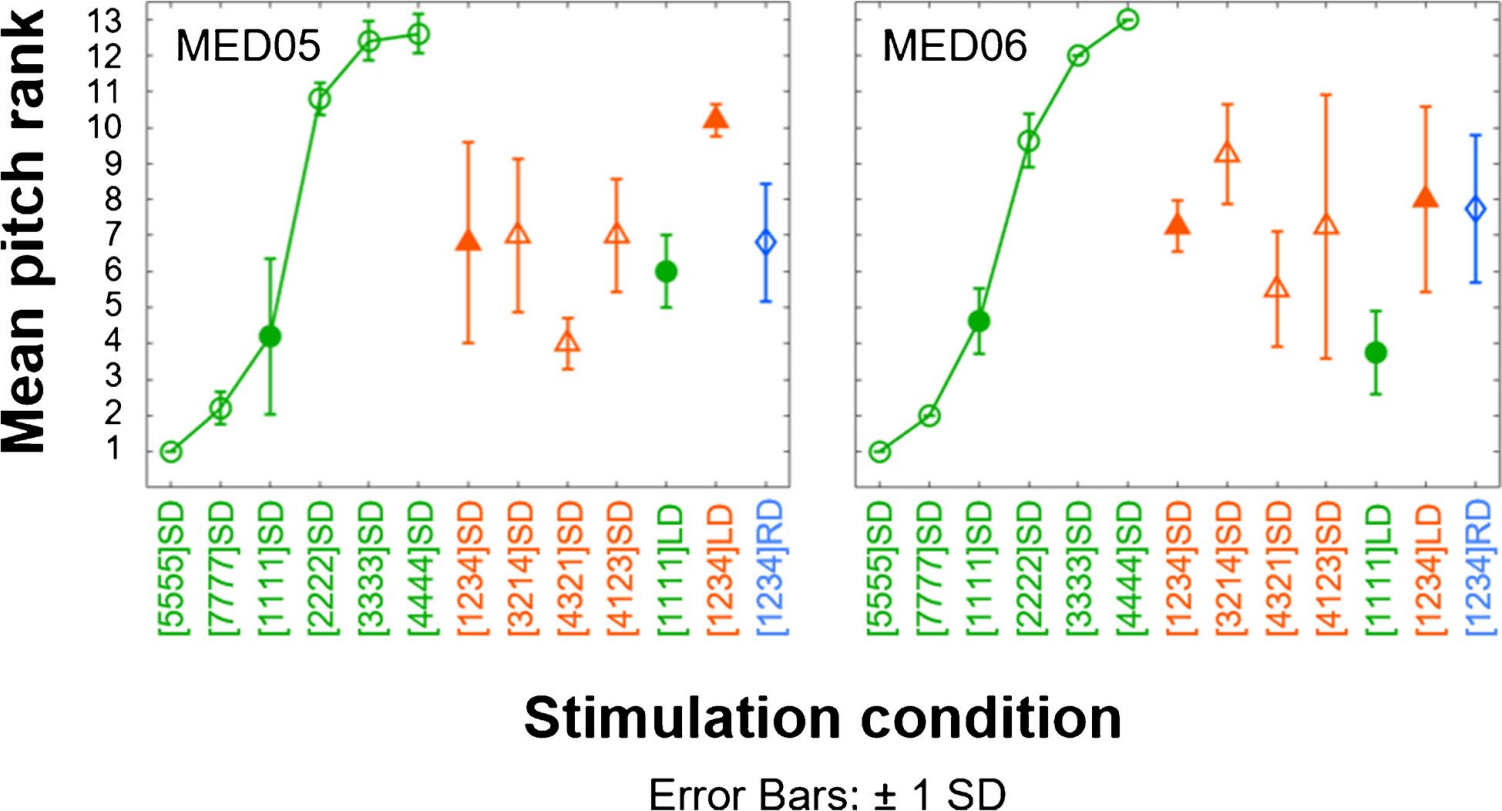
Mean rate-pitch ranks and standard deviations (SD) obtained from five runs of the midpoint comparison procedure for MED05 and from eight runs for MED06. The same-rate stimuli are presented by circles, of which the short-delay (SD) conditions are joined by lines. The mixed-rate conditions are presented by triangles. Filled markers indicate the SD to long delay (LD) comparisons. The reversed-delay (RD) condition is marked by a diamond

Finally, we note that, especially when the pitch of a stimulus is weak, pitch comparisons can be affected by the range of stimuli included in the comparison set. For example, Carlyon et al. [34] required listeners with single-sided deafness to make place-pitch comparisons between an electric pulse train presented to a CI electrode in one ear and to acoustic pulse trains bandpass-filtered into a range of different frequency regions in the contralateral NH ear. They reported that, for some combinations of listener and electrode, the acoustic pulse train judged equal in pitch to the CI pulse train fell in the middle of the range of acoustic stimuli included in a block of trials, such that changing the range of acoustic stimuli could substantially shift the “pitch match”. They suggested that in these conditions, the comparisons between the NH and CI pulse trains depended only on the pitch of the NH pulse train; when that pitch was higher than the middle of the range of acoustic pitches, then the CI pulse train was judged to have a pitch lower than the NH pulse train; the opposite bias would occur when the NH pitch was lower than the middle of the range of acoustic pitches heard. It is in principle possible that a similar phenomenon could occur here if only the short-delay same-rate stimuli had clear pitches; judgements involving one multi-rate stimulus and one same-rate stimulus might then have depended only on the pitch of the same-rate stimulus. To explain our results, the middle of the range of pitches heard in the same-rate stimuli would have to fall between that of [1111]SD and [2222]SD, and if correct, it would mean that the multi-rate stimuli had unclear pitches that did not necessarily lie between that of [1111]SD and [2222]SD. However, when we repeated the pitch-ranking experiment with listeners MED05 and MED06 and including an additional 2 same-rate conditions with rates of 50 and 71 pps on every channel, we found that the pitch rank for stimulus [1234]SD still generally fell between the ranks for [1111]SD and [2222]SD (Fig. 5), and with no evidence that the mixed-rate stimuli were matched to a lower pitch than in the main experiment. In addition, we replicated the finding of a lower pitch rank for the [4321]SD stimulus compared to the other mixed-rate conditions (orange symbols) for participant MED05, consistent with her pitch judgements being dominated by the pulse rate applied to electrode 4. Pitch ranks for the mixed-rate stimuli for participant MED05 were also broadly similar to those in the main experiment, albeit with large error bars for stimulus [4123]SD.

### Practical Implications and Suggestions

In the Introduction, we proposed two requirements for the effective transmission of pitch by FSP strategies. The results presented here, using simplified versions of the outputs of 4 FSP channels, showed that, broadly speaking, neither consideration was met. First, there was no evidence that listeners extracted the fundamental frequency from multiple channels; stimuli in which multiples of a 100-pps F0 were applied to the different electrodes produced variable pitch matches that did not correspond to that produced by an unambiguous stimulus ([1111]SD) in which all channels nearly-synchronously conveyed a 100-pps rate. Second, for most listeners, pitch ranks were affected by the temporal offsets between channels. In a real-life strategy, this suggests that pitch will be affected by factors such as reverberation and the group delays introduced by the analysis filters that may influence between-channel timing relationships. (The possible effect of the analysis filters could arise because, for a bandpass filter, the group delay varies as a function of the relationship between the input frequency and the filter’s centre frequency, and because this relationship will vary between the harmonics of a complex sound.) As a result, we believe that although an FSP strategy could do a good job of conveying the pitch of a sinusoid, the pitch of a complex harmonic sound is likely to be weak, vulnerable to features of the environment and (possibly) analysis filters, and does not correspond to the F0.

A *caveat* to the above conclusion is that, despite the absence of a strong, robust pitch, listeners might be able to identify the direction of pitch changes between successive notes (or vowels) or of dynamic F0 changes within a single sound. In terms of our nomenclature, this would correspond to a change from stimulus [1234]SD to e.g. [1.2, 2.4, 3.6, 4.8]SD causing a (weak) pitch to increase. Experiments with NH listeners have shown that listeners can indeed make fairly accurate sequential comparisons between two weak pitches. For example, although (as noted earlier) mixtures of inharmonically related pulse rates produce an unpleasant “crackle” percept without a clear pitch [44], listeners could identify the direction of changes in one of the rates in a mixture with an accuracy that was only moderately lower than when a single pulse train was presented. In addition, McPherson and McDermott [47] found that identification of the direction of a small F0 change between two temporally adjacent complex tones was equally good when those complex tones were inharmonic vs. harmonic; the discrimination of melodic contours also did not depend on harmonicity. However, a clear pitch was important for more-musical tasks (e.g. interval identification) and for the discrimination of F0 differences between notes separated by longer intervals [48].

We therefore believe that it is worth exploring alternative methods that, as far as possible, allow CI listeners to extract a robust pitch from harmonic complex sounds. One straightforward approach is inspired by the observation that the pitch ranks for the same-rate conditions were more reliable (smaller confidence intervals) than for the multi-rate conditions and increased monotonically with pulse rate up to about 300 pps [cf. 39, 40, 41, 49]. Combined with the effects of cross-channel timing differences, this suggests that presenting the same TFS to one or more electrodes might provide a more robust and stronger pitch percept than when the temporal pattern of stimulation differs across electrodes. This pattern could be derived for example from the output of a real-time F0 estimation algorithm and with the amplitude for each electrode determined by the envelope amplitude for that channel. Possible drawbacks could arise in noisy backgrounds and/or when more than one F0 was present, and because presenting pulses at the F0 rate may under-sample the envelope in the more-basal channels stimulated (which have wider analysis channels and hence contain faster modulations). However, even a robust perception of the pitch of single sounds in isolation may be considered an advance, and there may be a sweet spot in the trade-off between conveying F0 on more channels so as to maximise pitch salience and restricting that code to more apical low-frequency channels so as to minimize under-sampling of the envelope.

## Data Availability

The data and code that support the findings of this study will be made available upon request from the corresponding author.

## Code Availability

The code that analyses the data from this study is available from the corresponding author upon reasonable request.

## Declarations

### Artificial Intelligence (AI)

AI was not used for the generation of text or figures.
